# Fabrication and characterization of DNA-loaded zein nanospheres

**DOI:** 10.1186/1477-3155-10-44

**Published:** 2012-12-02

**Authors:** Mary C Regier, Jessica D Taylor, Tyler Borcyk, Yiqi Yang, Angela K Pannier

**Affiliations:** 1 Department of Biological Systems Engineering, University of Nebraska-Lincoln, 231 Chase Hall, Lincoln, NE 68583-0726, USA; 2 Department of Textiles, Clothing and Design, University of Nebraska-Lincoln, 231 Chase Hall, Lincoln, NE 68583-0726, USA

**Keywords:** Gene delivery, Nonviral, Zein, DNA, Nanoparticle, Oral delivery, Intramuscular injection

## Abstract

**Background:**

Particulates incorporating DNA are promising vehicles for gene delivery, with the ability to protect DNA and provide for controlled, localized, and sustained release and transfection. Zein, a hydrophobic protein from corn, is biocompatible and has properties that make it a promising candidate material for particulate delivery, including its ability to form nanospheres through coacervation and its insolubility under physiological conditions, making it capable of sustained release of encapsulated compounds. Due to the promise of this natural biomaterial for drug delivery, the objective of this study was to formulate zein nanospheres encapsulating DNA as the therapeutic compound, and to characterize size, charge, sustained release, cell cytotoxicity and cellular internalization of these particles.

**Results:**

Zein nanospheres encapsulating DNA were fabricated using a coacervation technique, without the use of harsh solvents or temperatures, resulting in the preservation of DNA integrity and particles with diameters that ranged from 157.8 ± 3.9 nm to 396.8 ± 16.1 nm, depending on zein to DNA ratio. DNA encapsulation efficiencies were maximized to 65.3 ± 1.9% with a maximum loading of 6.1 ± 0.2 mg DNA/g zein. The spheres protected encapsulated DNA from DNase I degradation and exhibited sustained plasmid release for at least 7 days, with minimal burst during the initial phase of release. Zein/DNA nanospheres demonstrated robust biocompatibility, cellular association, and internalization.

**Conclusions:**

This study represents the first report on the formation of zein particles encapsulating plasmid DNA, using simple fabrication techniques resulting in preservation of plasmid integrity and tunable sizes. DNA encapsulation efficiencies were maximized to acceptable levels at higher zein to DNA ratios, while loading was comparable to that of other hydrophilic compounds encapsulated in zein and that of DNA incorporated into PLGA nano- and microspheres. The hydrophobic nature of zein resulted in spheres capable of sustained release of plasmid DNA. Zein particles may be an excellent potential tool for the delivery of DNA with the ability to be fine-tuned for specific applications including oral gene delivery, intramuscular delivery, and in the fabrication of tissue engineering scaffolds.

## Background

Gene delivery, the introduction of exogenous DNA into cells with subsequent expression, is applicable to the fields of gene therapy [1], DNA vaccination [2], functional genomics and diagnostics [3], and tissue engineering [4]. Because of the technical and safety issues associated with viral gene delivery, the use of plasmid DNA (pDNA), which has lower immunogenicity, more flexibility in transgene capacity, and has the potential for industrial production, is an appealing alternative for gene transfer [5]. Delivery of pDNA can result in the expression of a therapeutic gene or induction of protective immunity [2,6]. Although the injection of naked pDNA can lead to transgene expression, the level and localization of expression are limited by rapid degradation by nucleases in the serum and clearance by the mononuclear phagocyte system [1]. Encapsulation of pDNA has the potential to improve *in vivo* response and transfection by shielding the CpG methylation patterns of pDNA from the immune system, shielding the plasmid from degradation by enzymes and low pH, increasing residence time, and providing a controlled release [7]. Nano- and microspheres incorporating pDNA, which administer pDNA in a controlled, sustained, and localized/targeted manner through the use of polymer systems that entrap pDNA and release it through hydrolytic or enzymatic mechanisms, have been applied to gene therapy, DNA vaccination, and tissue engineering [7,8].

For gene therapy and DNA vaccination applications, several routes of administration for DNA delivery systems are possible, including oral delivery and intramuscular injection. The oral route is perhaps the most appealing, due to its associated high patient compliance and convenience. The oral route has the additional advantages of presenting a large surface area of intestinal epithelium for transfection and allowing treatment of regional disorders by providing access to the luminal side of the intestine [9]. Oral gene delivery has the potential to treat diseases associated with the gastro-intestinal tract (GI-tract) [10] as well as systemic diseases [11], and can provide for systemic and mucosal immunity [12]. However, oral delivery of DNA is complicated by low pH in the stomach and DNases in the GI-tract [13], which degrade unprotected DNA. Particulates are considered a viable tool for the protection of DNA from the harsh environment of the stomach and intestine [14]. Previous studies have focused on poly(lactide-co-glycolide) (PLGA) and chitosan to form DNA-loaded nano- and microspheres for oral gene delivery [7,15-17]. While these delivery vehicles have been shown to elicit the production of a therapeutic or immune response inducing protein, the level of protein produced is often modest with high variability [16,18]. Furthermore, although these materials have provided a valuable proof of concept, their lack of sufficient efficacy suggests that new polymers need to be investigated.

The oral route of administration is not the only route of interest for gene therapy and DNA vaccination. The direct injection of DNA delivery systems into muscle has been investigated for DNA vaccination [19] and gene therapy [20]. Injection of DNA-loaded nanoparticles and microparticles into muscle primarily results in local transfection because the particles do not readily diffuse from the tissue. Transfection of muscle cells can elicit an immune response [19], can result in a physiological change in the injected muscle [21], or can serve as a depot releasing the encoded protein into circulation [20]. Particulate DNA delivery vehicles have been shown to increase the magnitude and duration of transgene expression compared to other delivery vehicles or naked DNA administered intramuscularly [7,22]. However, as for oral delivery of DNA-loaded particulates, lack of sufficient efficacy with intramuscular administration suggests that new polymers need to be investigated for DNA delivery.

Natural polymers have been applied to drug and gene delivery and tissue engineering applications, and have the advantages of providing innate degradability and bioactivity [23,24], but typically suffer from low mechanical properties, poor water stability, limited ability to be processed, and a relatively short release period compared to synthetic polymers [8,25]. However, some natural polymers derived from plants, including wheat gluten, glutenin, zein, soy protein, cellulose, and starch [26-28] overcome some of the shortcomings of natural polymers. Among the various plant proteins, zein, the prolamin or storage protein from corn, has properties that make it a promising candidate material for particulate delivery. Zein is composed of three fractions, which are defined by their molecular weight and solubility, including α (75-85% of total zein, 21–25 kDa and 10 kDa), β (10-15% of total zein , 17–18 kDa), and γ (5-10% of total zein, 27 kDa) zein [29]. More than 50% of the 225 amino acid residues [30] of zein are hydrophobic [31], including high percentages of leucine, proline, and alanine, which renders it insoluble under physiological conditions and capable of sustained release of encapsulated compounds [32]. Zein also has a high glutamine content [33,34], contributing polar, protonable side chains. With its amphiphilic character [35], the hydrophobic regions of zein can cause aggregation into colloidal particles, and the polar side chains allow for interaction with DNA. The surface charge of zein varies with the pH of the environment [36], with an isoelectric point of α-zein at pH 6.8 [31]. Degradation of zein occurs very slowly by hydrolysis but is accelerated by the action of enzymes [32] and has been shown to be especially well-suited for oral delivery [37-39]. Zein has been shown to be biocompatible and to have degradation products that can enhance cell proliferation [40]. Furthermore, part of the N-terminal region of γ-zein has been shown to interact with cell membranes and has served as a peptide carrier for drugs across cell membranes [41].

Nano- and microspheres composed of zein can be fabricated using a simple coacervation technique, which involves no harsh solvents or high temperatures. Zein microparticles have successfully been used to orally deliver ivermectin in a canine model [38] and desmopressin in a Phase I clinical trial [39]. However, to the best of our knowledge, there have been no reports on the fabrication of zein nanospheres encapsulating a large, charged and hydrophilic molecule, like pDNA. Due to the promise of this natural biomaterial for drug delivery, including its biocompatibility, promotion of cell proliferation, capability for sustained release, interaction with cell membranes, and its versatility to interact, encapsulate and protect cargo, the objective of this study was to formulate and characterize zein nanospheres encapsulating DNA as the therapeutic compound, and to perform investigations into their ability for sustained DNA release, as well as to characterize cell cytotoxicity and internalization.

## Results & discussion

### Sphere formation

Coacervation, the separation of solutions into colloidal systems with two liquid phases [42], was used to form spheres. This separation involves the formation of one phase rich in polymer (the coacervate) and another phase lacking polymer, which is brought about by the partial desolvation of a previously dissolved polymer [42-44]. Decreasing the ethanol concentration of dissolved zein solutions by the addition of aqueous solutions results in the necessary desolvation and the formation of a zein-rich nanosphere phase [45]. This method has been previously used to encapsulate a variety of drugs [46-49], however, to the best of our knowledge, no published studies have reported on the encapsulation of DNA in zein. In this current study, pDNA was included with the aqueous solution added to desolvate zein, which resulted in the encapsulation of pDNA within the zein nanosphere phase formed through coacervation. Encapsulation of hydrophilic pDNA within the hydrophobic zein spheres was enhanced by lowering the pH of the initial ethanolic zein solution, which resulted in a net positive charge on the protein due to its higher isoelectric point [31]. This change in charge promoted the electrostatic interaction of the zein and the negatively charged pDNA. During formation, suspensions of DNA-loaded zein nanospheres were free of visible aggregates during formation while blank spheres resulted in some visible aggregates (data not shown). Prior to centrifugation and collection of the spheres, the pH of the zein nanosphere suspensions was raised to 10 to lower the surface charge of the spheres to ~ −70 mV (a value sufficiently far from zero), resulting in repulsive forces between spheres to prevent irreversible aggregation of the spheres after centrifugation, and allowing for resuspension of the pelleted spheres for further analysis and delivery to cells. This sphere formation, centrifugation, and resuspenison procedure allowed for the simple and repeatable production of pDNA-loaded nanospheres, which were then characterized.

### Characterization

#### SEM

**Figure 1:**
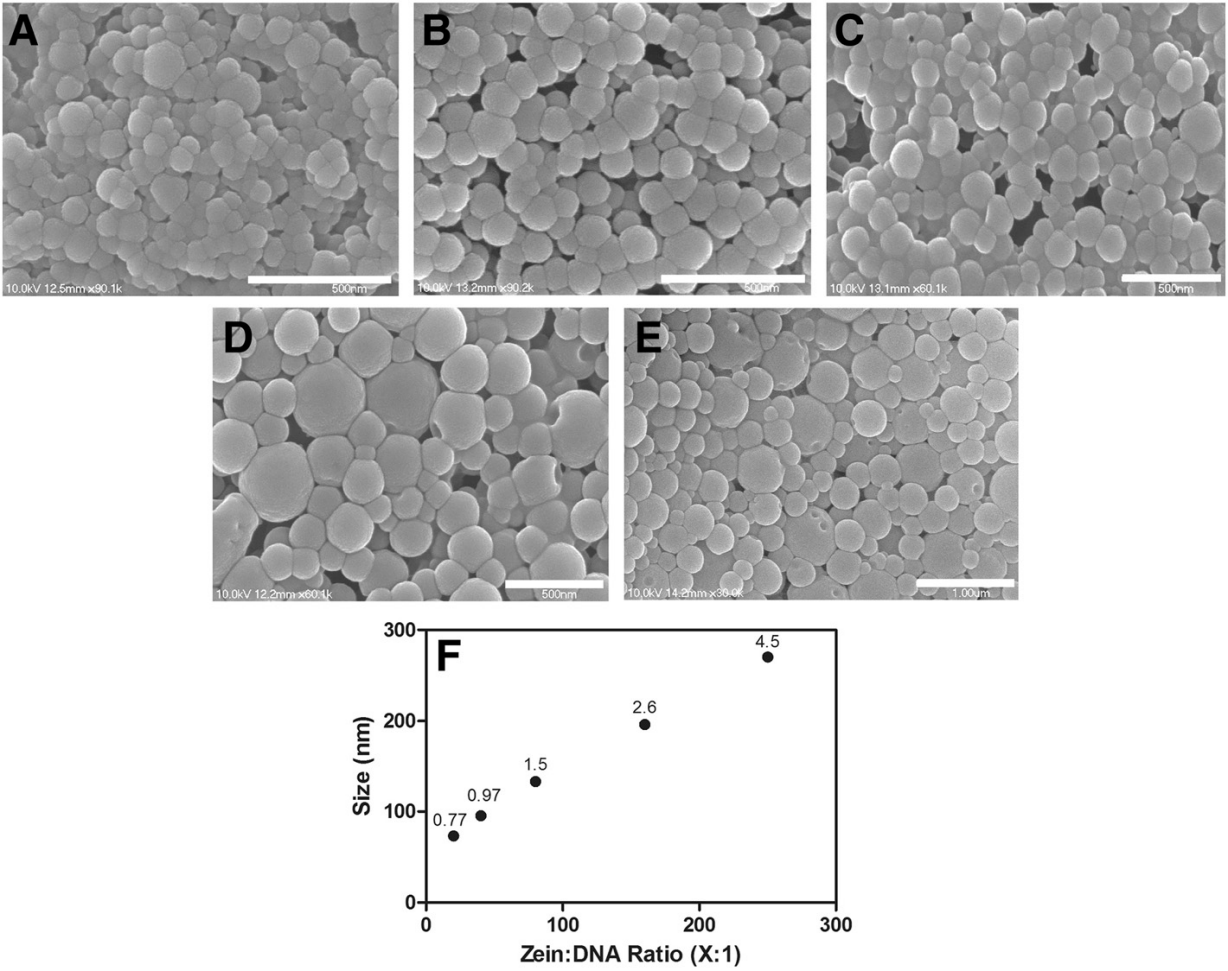
SEM of zein-DNA nanospheres formed at 20:1 (A, 90 k magnification, scale bar 500 nm), 40:1 (B, 90 k, scale bar 500 nm), 80:1 (C, 60 k, scale bar 500 nm), 160:1(D, 60 k, scale bar 500 nm), 250:1 (E, 30 k, scale bar 1 μm) zein:DNA ratios; average diameter for each ratio measured from SEM images (F), reported as mean ± standard error of the mean.

The morphology and size of the zein/DNA nanospheres were characterized using SEM. SEM images confirmed the spherical nature of the nanospheres with smooth surfaces (Figure 1) at all ratios of zein to pDNA. Average sizes measured from SEM images were 73.0 ± 0.8 nm (Figure 1A), 95.4 ± 1.0 nm (Figure 1B), 133.2 ± 1.5 nm (Figure 1C), 196.1 ± 2.6 nm (Figure 1D), and 270 ± 4.5 (Figure 1E) for 20:1, 40:1, 80:1, 160:1 and 250:1 for zein:DNA spheres, respectively (Figure 1F). The spheres prepared in this study increased in size linearly as the zein to pDNA ratio increased, but were relatively small compared to zein nanospheres prepared by similar methods, which have typically been in the 0.5 - 2 μm [48], 0.3 -1.2 μm [49], and 1–1.7 μm [50] range. The smaller size of the DNA-loaded spheres could be attributed to electrostatic compaction of the particles or as a result of the precipitation step, when the pDNA may have formed numerous small particles, which could induce zein to form many small zein/DNA nanoparticles during coacervation. The increase in sphere size with an increase in the ratio of zein to incorporated compound has been reported elsewhere [45]. This increase in sphere size could be due to a lower mass of pDNA associated with the surface, which may result in an increase in interfacial tension between the hydrophobic sphere surface and the aqueous medium, leading to a greater radius of curvature [45]. These data suggest that the amount of pDNA present affects the nucleation and growth of zein/DNA nanospheres and that size can be controlled by adjusting the zein to pDNA ratio.

#### DLS

**Figure 2:**
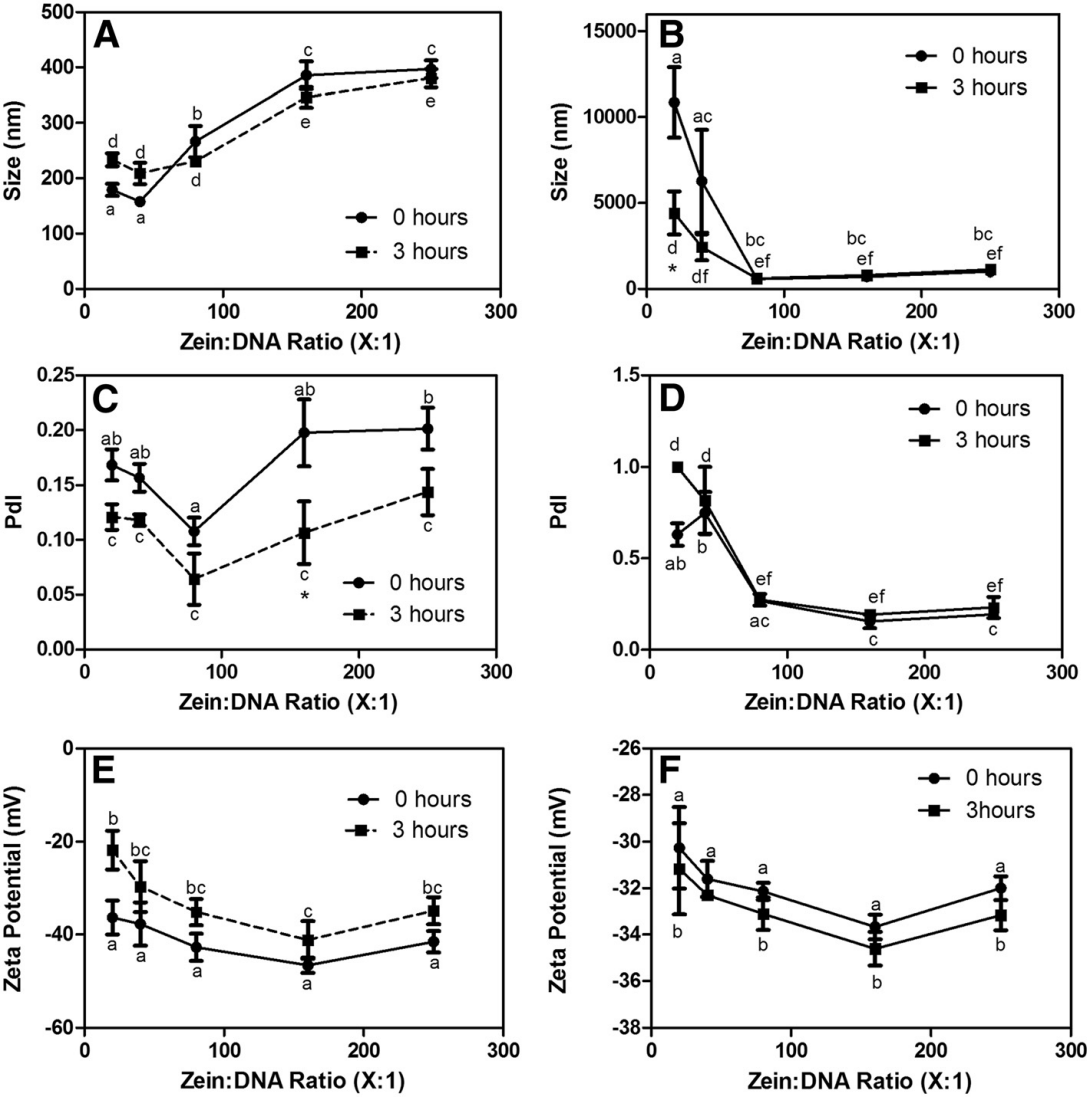
**Size, PdI, and zeta potential for zein/DNA nanospheres formed at various zein to DNA ratios measured directly after resuspension (0 hours) and three hours after resuspension (3 hours) in wate (A, C, E) or PBS (B, D, F). **Data points labeled with the same letter are not significantly different, while those labeled with asterisks vary significantly (p < 0.05) between the zero hour and 3 hour measurements. All data are reported as mean ± standard error of the mean, with n = 6.

Size and zeta potential were measured to further characterize and assess the stability of zein/DNA nanospheres. Immediately after resuspension, the hydrodynamic diameters of zein/DNA nanospheres measured in water increased with increasing zein:DNA ratio for 40:1, 80:1, and 160:1 zein:DNA ratios (157.8 ± 3.9 nm, 266.5 ± 28.2 nm, and 385.6 ± 25.0 nm, respectively), similar to the trend observed in SEM images; however no significant differences in size existed between 20:1 (178.9 ± 10.9 nm) and 40:1 and between 160:1 and 250:1 (396.8 ± 16.1 nm) ratios for spheres measured by DLS (Figure 2A). The average measured diameter from DLS analysis was found to be greater than the corresponding diameter measured by SEM for all ratios [51], which may be attributed to the presence of residual solvent in the spheres that had not been dried, and/or swelling, which has been reported for zein fibers in an aqueous medium [52] due to zein’s hygroscopic character [53]. Zein/DNA nanospheres prepared at zein:DNA ratios of 80:1 or higher were stable in water over three hours, and the 20:1 and 40:1 zein:DNA nanospheres exhibited a slight increase in diameter over the three hours of incubation in water, indicating the possibility of minimal aggregation at these ratios. In general PdIs varied between 0.2 and 0.1 demonstrating uniformity of the spheres (Figure 2C).

Spheres were characterized in PBS to assess the stability of spheres in a solution with physiological salt concentration and pH. In this environment, size measurements above 10 μm (which is beyond the detection limit of the DLS instrument and thus should not be considered accurate) and PdI values over 0.5 for both 20:1 and 40:1 zein:DNA spheres align with the observation of aggregation at these ratios (Figure 2B, D). For 80:1 and higher zein:DNA ratios, spheres did not aggregate in PBS and measured sizes were much lower than those of 20:1 and 40:1 particles in PBS, but were still higher than those measured in water. These larger sizes, between 740 and 1120 nm, measured in PBS were attributed to salt induced aggregation [54], which was subsequently verified by measuring size and zeta potential and noting the degree of resuspension and aggregation in solutions with varying salt concentrations (Additional file 1: Figure S1). Spheres formulated at the higher zein:DNA ratios showed no or little change in size over three hours in both water and PBS. The zeta potentials of the spheres formed at the various zein to DNA ratios were relatively uniform in both water and PBS, with values distributed between −20 and −50 mV (Figure 2E, F). Negative values were expected as zein has an isoelectric point of 6.8 [31] and DNA is also negatively charged at physiological pH.

Overall, the characteristics of the zein/DNA nanospheres formulated in this study suggest that the spheres could be well-suited for oral administration, particularly for DNA vaccination, as particles less than 10 μm in diameter are taken up transparacellularly by Peyer’s patches in the intestine [55], a target for DNA-encoding antigens because of their proximity to gut-associated lymphoid tissue [56]. Stabilization of the 20:1 and 40:1 particles could lend them to oral administration for gene therapy where widespread transfection is desired or for intramuscular injection, since particles less than 200 nm in diameter are taken up by endocytosis [55]. While the negative surface charge measured for the zein/DNA spheres may prevent favorable interactions with the negatively charged cell membrane, a negative surface charge may impart mucoadhesive properties to the particles, which could increase intestinal residence time [57], further enhancing the ability of these particles to be used for oral administration of DNA therapeutics. In addition, zein/DNA nanospheres could be used for intramuscular injection, for DNA vaccination [19], to deliver a therapeutic gene targeted to muscle tissue [21], or for systemic gene therapy [20]. Although the efficacy of the zein/DNA nanospheres formulated as described in this study could benefit from stabilization under physiological conditions and alteration of the surface charge, the spheres have appropriate size and uniformity for delivery of genes.

#### Encapsulation & loading

**Figure 3:**
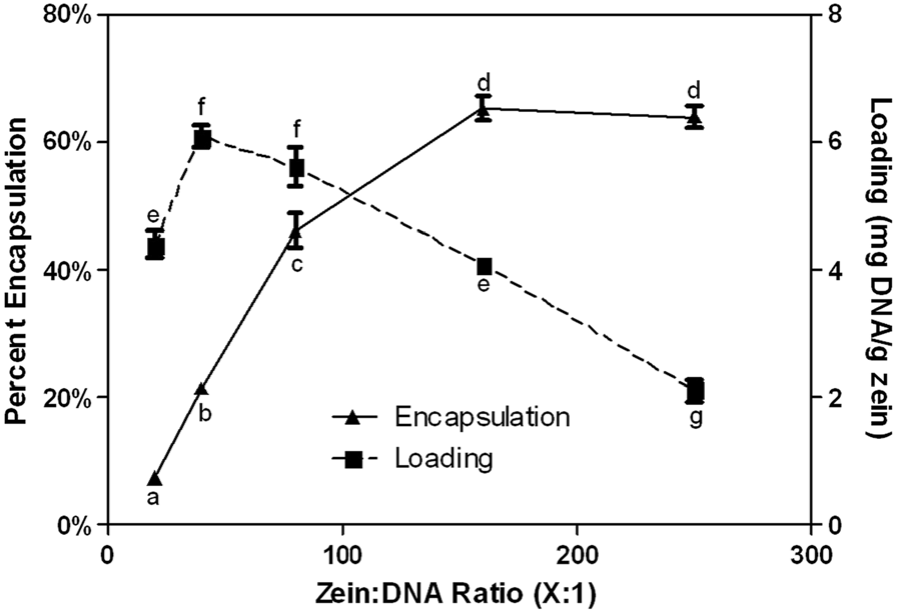
**Percent encapsulation and loading of nanospheres prepared at various zein to DNA ratios.** Data labeled with the same letter are not significantly different. Data are reported as mean ± standard error of the mean, with n = 6.

To determine the efficiency of DNA encapsulation for the method used in this study, percent encapsulation and loading were measured. Similar to sphere size, encapsulation and loading of DNA in zein nanospheres was dependent on the ratio of zein to DNA (Figure 3). Percent of DNA encapsulation increased with the ratio of zein:DNA up to 160:1 where it reached its maximum of 65.3% ± 1.9%. Loading was highest with 6.1 ± 0.2 mg DNA/g zein for spheres with a zein:DNA ratio of 40:1. The encapsulation of hydrophilic drugs in zein is more difficult to achieve than the encapsulation of hydrophobic drugs presumably due to hydrophilic/hydrophobic interactions. The pDNA loading reported here is typical for the encapsulation of relatively hydrophilic drugs in zein nano- and microspheres [47,48] while higher loading (up to 190 mg drug/g zein) has been attained by conjugating the hydrophilic drug to zein prior to particle formation [46]. Furthermore, the pDNA loading achieved in this study is comparable to that achieved with most PLGA nano- and microspheres [58], but is much lower than DNA loading into chitosan/DNA nanoparticles [59,60]. These data indicate that DNA loading can be tailored by changing the zein:DNA ratio, and preliminary results indicate that increased loading may be produced by varying the pH and ethanol concentration of the solution used to dissolve zein (data not shown). Because nanospheres formed at a zein to DNA ratio of 80:1 did not aggregate in PBS and had relatively high encapsulation efficiency and loading, this ratio was chosen for further characterization and cell studies.

#### DNA integrity

**Figure 4:**
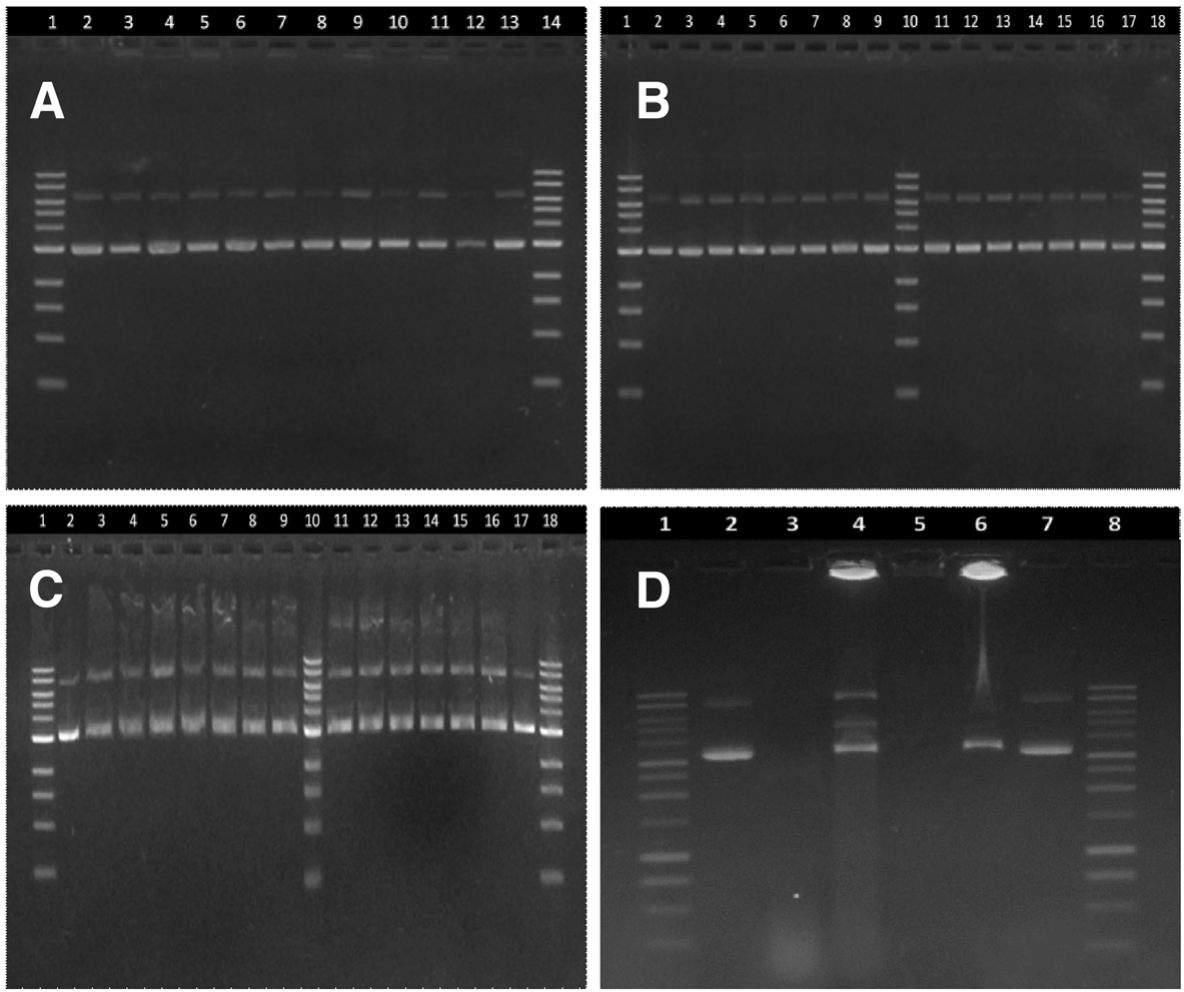
**Agarose gel electrophoresis images of extracted samples for spheres made at various zein to DNA ratios (A): lane 1, ladder; lane 2, stock DNA; lane 3, 20:1 spheres; lane 4, 20:1 supernatant; lane 5, 40:1 spheres; lane 6, 40:1 supernatant; lane 7, 80:1 spheres; lane 8, 80:1 supernatant; lane 9, 160:1 spheres; lane 10, 160:1 supernatant; lane 11, 250:1 spheres; lane 12, 250:1 supernatant; lane 13, stock DNA; lane 14 ladder. **Agarose gel image of DNA extracted from spheres (**B**) and supernatants (**C**) at various time points in the PBS release study: lane 1, ladder; lane 2, stock DNA; lane 3, 0 hr; lane 4, 1 hr; lane 5, 3 hr; lane 6, 6 hr; lane 7, 9 hr; lane 8, 12 hr; lane 9, 24 hr; lane 10, ladder; lane 11, 48 hr; lane 12, 72 hr; lane 13, 96 hr; lane 14, 120 hr; lane 15, 144 hr; lane 16, 168 hr; lane 17, stock DNA; lane 18, ladder. Agarose gel electrophoresis images of pDNA in DNase I degradation assay : lane 1, ladder; lane 2, stock DNA; lane 3, Naked DNA + DNase I; lane 4, 80:1 spheres + DNase I; lane 5, blank spheres (zein with no DNA); lane 6, 80:1 spheres ; lane 7, stock DNA; lane 8, ladder.

DNA integrity is necessary for efficient gene delivery, specifically maintenance of the supercoiled conformation; thus integrity measurements were made to ensure that encapsulation of the pDNA within the zein spheres did not damage the pDNA structure. Agarose gel electrophoresis revealed that pDNA was not damaged by encapsulation under the conditions used in this study (Figure 4A). pDNA integrity was also maintained in spheres during seven days of incubation in PBS for the release studies, as indicated by a supercoiled to nicked ratio similar to that of stock pDNA (Figure 4B). Although the nicked band appears to be larger in release samples than in the stock pDNA, there does not appear to be an increase in the ratio of nicked to supercoiled DNA over the seven day span of the release study (Figure 4C). While the stability of the pDNA in and released from zein particles is critical for both oral delivery and intramuscular applications, it also suggests that these zein/DNA nanospheres could be used for the fabrication of scaffolds with sustained gene delivery in tissue engineering [4], in that this stability is superior to that of pDNA encapsulated in PLGA microspheres, which produce degradation products that lower the local pH and can degrade pDNA [61]. Finally, the ability of zein spheres to protect encapsulated pDNA against nucleases was evaluated with a DNase I assay. Zein nanospheres showed protection of encapsulated pDNA from DNase I degradation, with pDNA concentrated at the top of the gel and unable to migrate from the loading well, due to its strong interaction with zein (Figure 4D). The presence of some degradation products can be attributed to surface-associated pDNA on the zein spheres (evident in lane 4), but the majority of the pDNA remained intact and encapsulated.

### Release study

**Figure 5:**
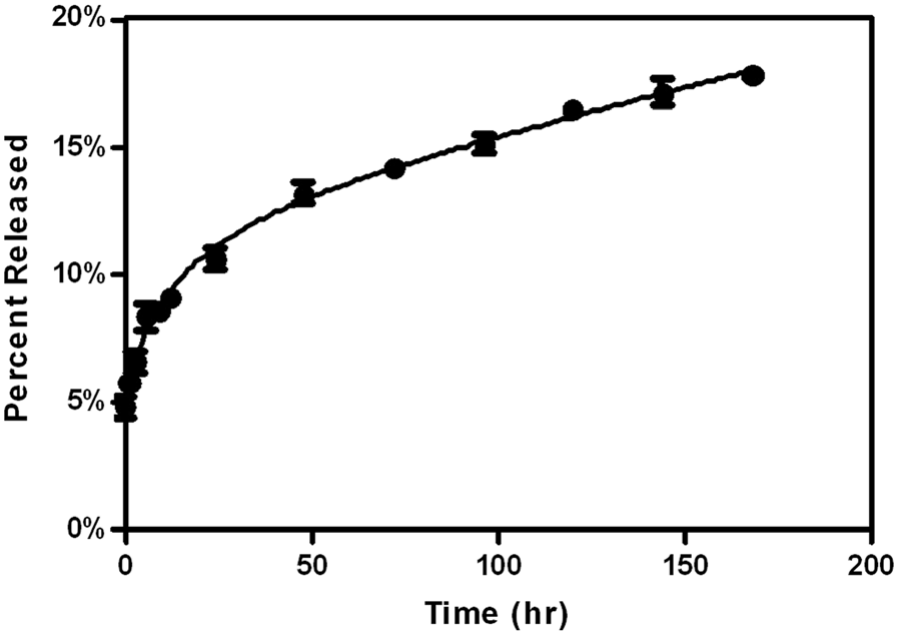
**Release of DNA from 80**:**1 zein spheres incubated in PBS at 37°C over 7 days. **Data reported as mean ± standard error of the mean, with n = 5.

pDNA release from zein nanospheres was measured in a standard PBS buffer. Nanospheres formulated at the 80:1 zein to DNA ratio released encapsulated pDNA relatively rapidly for the first 12 hours of the PBS release study (Figure 5). This period of fast release was followed by a near zero order release profile for the remainder of the study. After seven days in PBS only 17.8 ± 0.2% of the encapsulated pDNA was released. Sustained release of encapsulated drug in PBS has previously been demonstrated for zein nano- and microspheres [45,62,63]. These results indicate that if breakdown occurs by hydrolysis, it does so slowly, allowing for the release DNA in a sustained manner over at least one week and likely much longer, which is critical for intramuscular injection and tissue engineering applications. For these applications, sustained release of pDNA over time allows for transfection to a large number of cells at a localized site for the enhancement of therapeutic effect [22] or tissue development [4]. In addition, internalized particles could provide intracellular sustained release of pDNA. The observed sustained release for these zein/DNA spheres also verifies that pDNA is encapsulated within the particles rather than being absorbed to the surface.

### Cell studies

**Figure 6:**
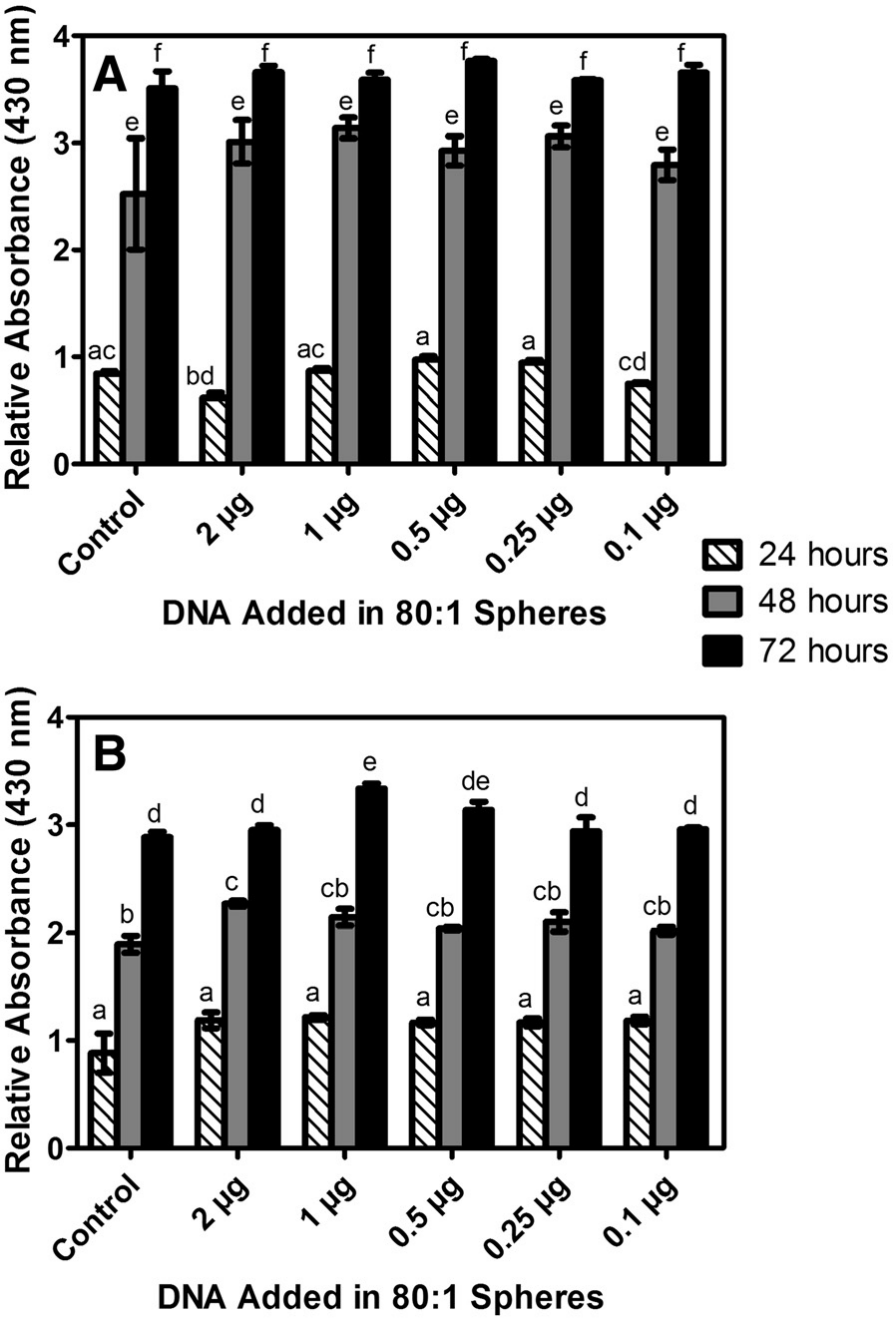
**Cytotoxicity of zein**-**DNA nanospheres quantified by WST**-**1 assay for (A) HEK 293T and (B) Caco-2 cells as a function of time and DNA dose. **Control condition represents cells without the addition of particles. Data points labeled with the same letter are not significantly different (p < 0.05). Data reported as mean ± standard error of the mean, with n = 3.

**Figure 7:**
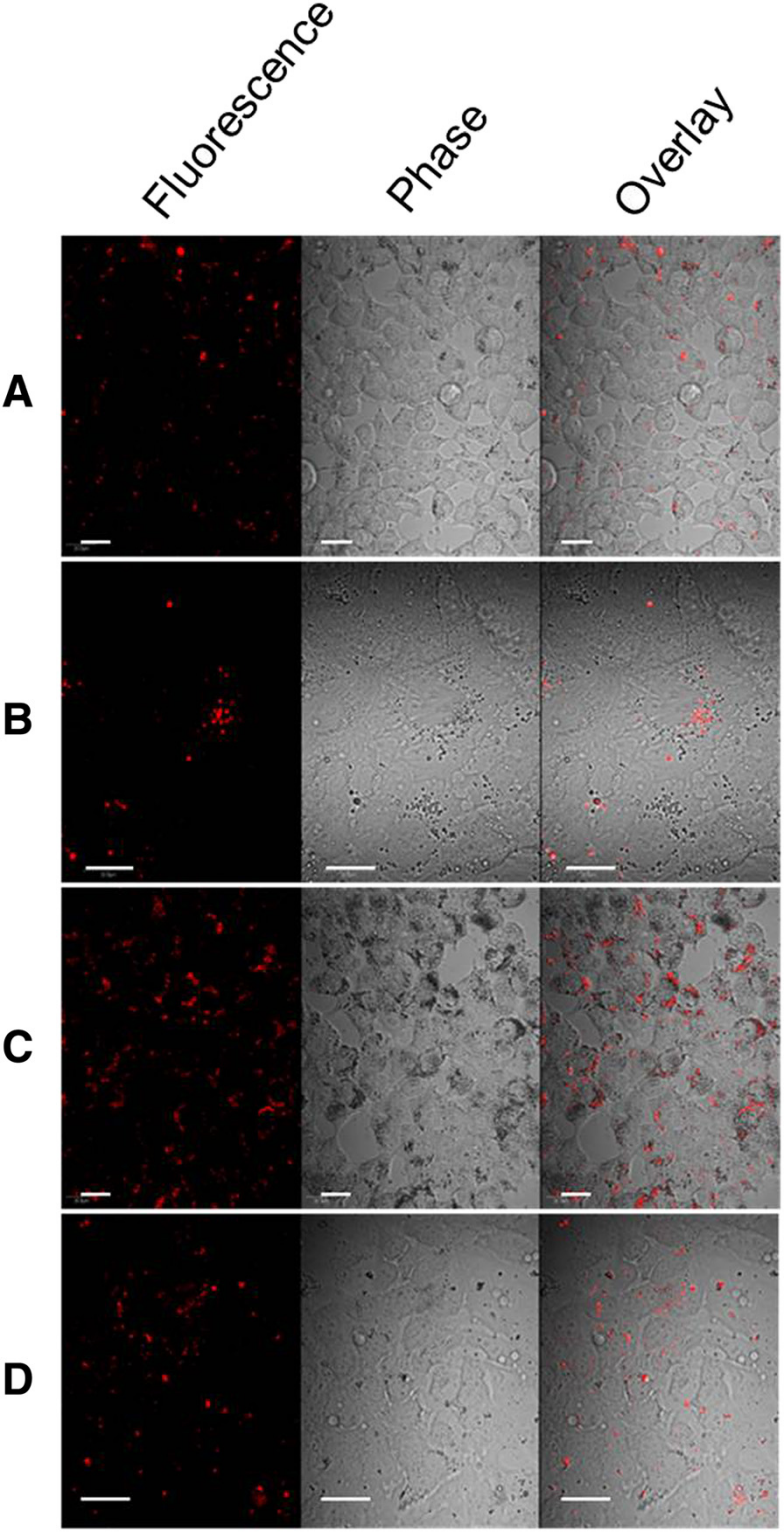
**Confocal images of HEK 293T cells with associated fluorescent 80:1 spheres (A, 600X magnification, 0.1 μg DNA/well) and 250:1 spheres (C, 600X, 0.1 μg DNA/well), and Caco-2 cells with associated 80:1 spheres (B, 600X, 0.1 μg DNA/well) and 250:1 spheres (D, 600X, 0.1 μg DNA/well). **It should be noted that not all of the particles fluoresced in the images as some were out of the focal plane of the microscope. Scale bars represent 20 μm.

**Figure 8:**
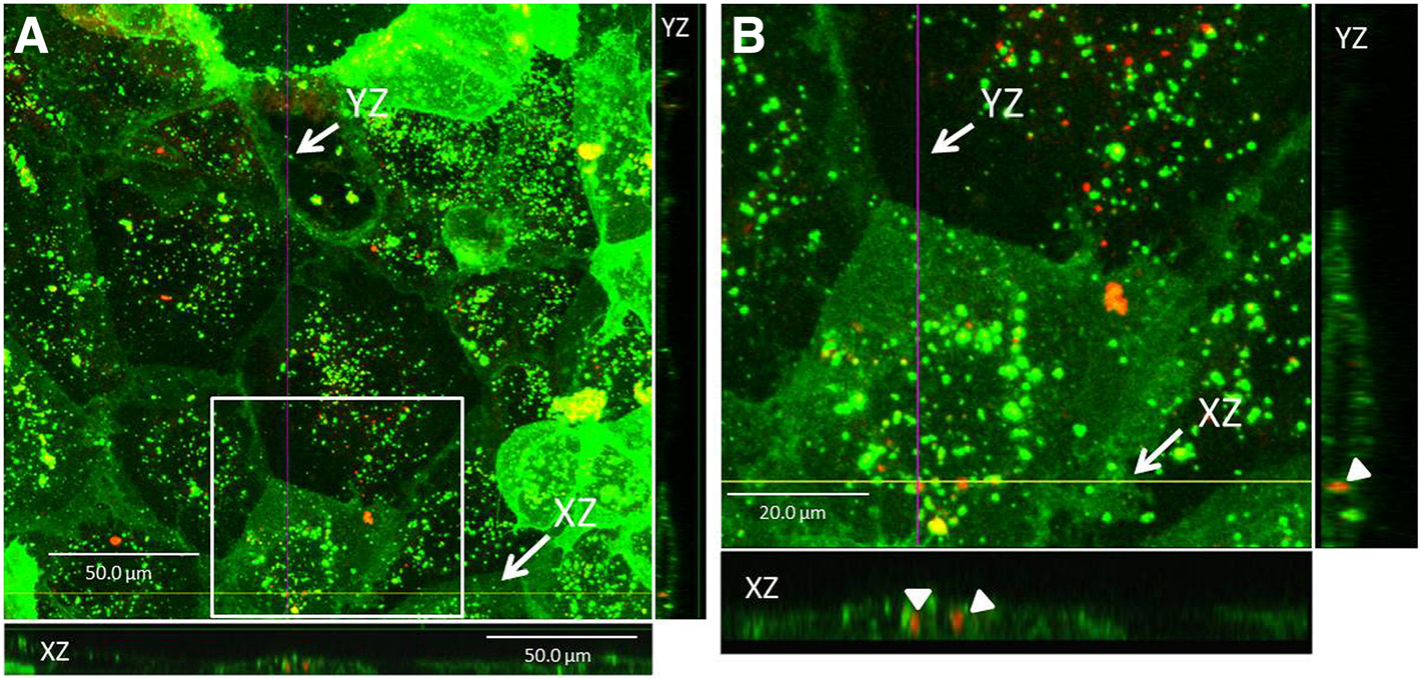
**Analysis of sphere internalization within Caco-2 cells, with DiO membrane staining, using merged fluorescence confocal images of entire z-stack with orthogonal views, XZ and YZ (A) and a digital magnification of the area outlined by a white square in A (B). **Punctate green staining within cells are indicative of endosomes. Filled arrows indicate internalized, autofluorescent nanospheres. Scale bars in A and B represent 50 and 20 μm, respectively.

Cell studies were conducted to determine biocompatibility, cellular association and internalization of the zein/DNA particles. Biocompatibility of the zein/DNA spheres with Caco-2 and HEK 293T cell lines was measured using a WST-1 assay. For HEK 293T cells, absorbance values at a wavelength of 430 nm after 24 hours only showed statistically significant differences between cells that were dosed with 2 μg zein/DNA particles and the control (cell only) condition, but at 48 and 72 hours, all zein/DNA conditions resulted in absorbance values that were not statistically significantly different from the control condition, indicating no cytotoxicity (Figure 6A). For Caco-2 cells, absorbance values at a wavelength of 430 nm at all times points (24, 48 and 72 hours) showed no statistically significant differences between cells that were dosed with zein/DNA particles and the control (cell only) condition, except for two concentrations of zein/DNA nanoparticles (2 μg and 1 μg of DNA at 48 and 72 hours, respectively), where absorbance values were significantly increased (p < 0.05) over the control condition, suggesting an increase in cell proliferation, similar to previous studies that have shown that the degradation products of zein can enhance cell proliferation [40]. For both cell types there was a lack of a dose response in that the addition of more spheres did not result in increased cytotoxicity as measured by the WST-1 assay, indicating that even at high doses the zein/DNA spheres were biocompatible. In addition, morphology appeared normal for both cell types incubated with zein/DNA nanospheres (Figure 7 and data not shown). These results demonstrate that the spheres in this current study possess biocompatibility with cells. For confocal imaging, nanospheres were not labeled as zein was observed to autofluoresce at multiple wavelengths of excitation and emission. For example, there was a linear relationship between zein concentration and fluorescence for excitation wavelengths of 365–395 nm and 465–485 nm, indicating that zein autofluoresces under these conditions (Additional file 2: Figure S2). Confocal images of HEK 293T and Caco-2 cells with added particles revealed a high degree of particle association with both cell types (Figure 7), with no noticeable changes in cell morphology. Fluorescent nanospheres were found to concentrate on cells more than in the intercellular spaces of the culture slides, indicating favorable cell-sphere interactions. To further assess internalization of the zein nanospheres within the Caco-2 cells, cellular membranes were stained with Vybrant DiO and then confocal imaging was used to visualize both the membrane and sphere locations (Figure 8). Sphere internalization was observed in over half of the cells in any particular image (Figure 8A). Further analysis of individual cells that were shown to be associated with spheres, through examination of individual z planes within the plane of the cell, confirmed internalization of the spheres. For instance in Figure 8B, two zein particles in the XZ plane are clearly internalized, which is further confirmed in the YZ plane. Punctate green staining within cells is indicative of endosomes. While transfection was not observed within the time course of these internalization studies due to slow release profile of DNA from the spheres, these results suggest that DNA/zein nanospheres can enter cells and thus be used to deliver DNA when properly optimized.

## Conclusions

In this work, zein, a natural protein, was investigated as a possible material for the formation of spheres encapsulating pDNA for gene delivery. Zein is a protein that is insoluble under physiological conditions, which can allow for sustained release of encapsulated compounds and enable zein to act more like a hydrophobic polymer (i.e. PLGA). Zein is biocompatible and has degradation products that can enhance cell proliferation [40]. Furthermore, part of the N-terminal region of γ-zein has been shown to interact with cell membranes and has served as a peptide carrier for drugs across cell membranes [41]. Zein is commonly used in pharmaceutical tableting and coating, and is considered most promising for applications in edible and biodegradable packaging and coatings as well as biomedical applications [64]. Zein has also shown great potential in the field of drug delivery [40,65,66], but previous studies have focused on small molecule, hydrophobic drugs. However, properties of zein make it well-suited for the development of nonviral gene delivery systems. Here, zein nanospheres encapsulating DNA were formed using a simple coacervation method, without the use of harsh solvents or temperatures, resulting in the preservation of pDNA integrity and tunable sizes. DNA encapsulation efficiencies were maximized to acceptable levels at higher zein to DNA ratios, while loading was comparable to that of other hydrophilic compounds encapsulated in zein and that of DNA incorporated into PLGA nano- and microspheres. The spheres were able to protect encapsulated DNA against degradation by DNAse I, and the hydrophobic nature of zein resulted in spheres capable of sustained release of plasmid DNA. Zein-DNA nanospheres showed biocompatibility with cells, and microscopy of cells with nano/microspheres indicated that the particles were highly associated with cells and were internalized. Future work will include modifications to improve cellular uptake and transfection. Zein appears to be an excellent potential tool for the delivery of DNA with the ability to be fine-tuned for specific applications including oral gene delivery, intramuscular injection, and in the fabrication of tissue engineering scaffolds.

## Materials and methods

### Plasmid preparation

All experiments used pDsRed2-N1 (Clontech, Mountain View, CA), a plasmid encoding for the red fluorescent protein, DsRed2. The plasmid was purified from bacteria culture using Qiagen (Valencia, CA) reagents and stored in Tris-EDTA (TE) buffer solution (10 mM Tris, 1 mM EDTA, pH 7.4) at −20°C. Only plasmids with purity of 1.8 or better measured by 260/280 ratio (Nanodrop 2000 Spectrophotometer, Thermo Scientific, Waltham, MA) were used.

### Sphere preparation

Zein spheres were formed by the coacervation technique. Zein (Freeman Industries LLC, Tuckahoe, New York) was first dissolved in 70% ethanol at pH 3 (pH adjusted with 1 M hydrochloric acid) forming a 1% w/v zein solution. Subsequently, 1 mL of 100% ethanol pH 3 was added to 1 mL of the 1% zein solution. A total of 1 mL of DsRed2 plasmid DNA (1 mg/mL in TE buffer) and TE buffer were added, followed by the dropwise addition of 10 mL ddH_2_O (18.2 mΩ-cm) while vortexing. Spheres were formed by coacervation during the addition of the ddH_2_O. The zein to DNA ratio was determined by the amount of DNA solution added and ranged from 20:1 to 250:1. Blank spheres were formed without DNA (TE buffer substituted for DNA solution). The pH of the resultant sphere suspension was increased to 10 by the addition of 1 M NaOH so that the spheres could be resuspended, as this pH induced sufficient surface charge on the spheres to prevent aggregation during centrifugation [67-69]. Spheres were then pelleted by centrifugation at 10,000 g for 1 hour at room temperature. The supernatant was removed and spheres were resuspended in ddH_2_O.

### Sphere characterization

#### Field-emission scanning electron microscopy

Scanning electron microscopy (SEM, S4700 Field-Emission SEM, Hitachi, Japan) was used to image and subsequently analyze sphere size and morphology. For SEM imaging, spheres were resuspended in 2 mL of ddH_2_O and lyophilized. The lyophilized powder was placed in a thin layer on carbon tape (Electron Microscopy Sciences, Hatfield, PA) and mounted for imaging. Spheres were then sputter coated with chromium and imaged at 10 kV and varying magnifications. Three micrographs for each ratio were analyzed using Image J (NIH, Bethesda, MD) to determine average sphere diameter.

#### Dynamic light scattering

A dynamic light scattering (DLS) analyzer (Zetasizer Nano ZS90, Malvern Instruments Ltd, UK) was used to measure the mean diameter and particle size distribution of spheres after dilution in either 1X Dulbecco’s phosphate buffered saline (PBS, Invitrogen, Carlsbad, CA) or ddH_2_O. For dilution in PBS, spheres were first resuspended in 2 mL of water and then diluted further with 1X PBS due to the inability of spheres to initially be completely resuspended in PBS. Intensity mean diameter was used to express average particle size. The same instrument was used to measure the zeta potential of the spheres by a combination of laser Doppler velocimetry and phase analysis light scattering. Sphere suspensions above were likewise diluted in 1X PBS or ddH_2_O before measurement. Size, zeta potential and the polydispersity index (PdI) were measured directly after resuspension and 3 hours after resuspension for each sample to assess stability. To determine the cause of aggregation of the spheres in PBS and the inability of pelleted spheres to be resuspended in PBS, sphere size and zeta potential were similarly measured at various salt concentrations. The spheres were resuspended and diluted in 1X PBS or ddH_2_O with NaCl concentrations of 300, 150, 50 and 10 mM. Size and zeta potential were measured before centrifugation and again after centrifugation and resuspension. The degree of resuspension was noted for all conditions.

#### Encapsulation & loading

pDNA encapsulation within the spheres was quantified using a standard phenol/chloroform extraction followed by fluorescent staining with Hoechst 33258 (Invitrogen). After sphere preparation and centrifugation, the supernatant was removed to a separate conical tube and pelleted spheres were resuspended in 5 mL ddH_2_O. An equal volume of phenol/chloroform/isoamyl alcohol 25:24:1 (Fisher BioReagents, Fair Lawn, NJ) was added to both the sphere and the supernatant solutions and these solutions were vortexed. The solutions were centrifuged at 10,000 g for 15 minutes at 4°C and then the top, aqueous layer was removed to separate tubes, to which 0.5 volume of chloroform (Fisher Chemical, Fair Lawn, NJ) was added, followed by vortexing. Solutions were again centrifuged at 10,000 g for 15 minutes at 4°C and the top, aqueous layer was then removed to separate tubes, which were centrifuged at 10,000 g for 15 minutes at 4°C to separate any residual chloroform from the aqueous layer. Any organic layer present was removed from the bottom of the tubes. The volumes of the aqueous DNA solutions were measured. Samples extracted from spheres and supernatants were then diluted appropriately in 1X TNE buffer (10 mM Tris; 0.2 M NaCl; 1 mM EDTA; pH 7.4), mixed with one volume Hoechst dye solution (200 ng/mL) and fluorescence was measured by a fluorometer (Modulus Luminometer/Fluorometer, Turner Biosystems, Sunnyvale, CA) after 5 minutes incubation at room temperature. A Hoechst standard curve was produced by graphing raw fluorescence versus DNA concentration for various dilutions of the stock DNA solution. The standard curve was then used to quantify the amount of DNA in the sphere and supernatant extraction solutions, using fluorescence measurements that were first normalized to the fluorescence of blank sphere extraction solutions. The mass balance for DNA was closed between 80% and 101% as measured by recovery:

(1) $$\text{Recovery}=\frac{\text{Mass of DNA in Sphere Extract+Mass of DNA in Supernatant Extract}}{\text{Mass of DNA Added}}\times 100\%$$

Percent encapsulation was calculated as:

(2) $$\text{Percent Encapsulation}=\frac{\text{Mass of DNA in Sphere Extract}}{\text{Mass of DNA Added}}\times 100\%$$

For each sphere preparation ratio of zein to DNA, the mass of DNA in spheres per mass of spheres was calculated, using measurements of DNA encapsulation described above and mass of lyophilized spheres for each ratio, as:

(3) $$\text{Loading}=\frac{\text{Mass of DNA in Sphere Extract (mg})}{\text{Mass of Spheres (g})}$$

#### DNA integrity

Sphere extraction solutions and release samples (as described below) were analyzed for plasmid integrity, as was the ability of the spheres to protect encapsulated pDNA from endonucleases. For the latter, naked pDNA (10 μg) and DNA-loaded zein spheres (equivalent to 10 μg of pDNA) were treated with 7.5 U of DNase I (Promega, Madison, WI) and incubated for 15 min at 37°C. The reaction was stopped by adding 50 mM Ethylenediaminetetraacetic acid (EDTA) and incubating at 65°C for 10 min. The integrity of the pDNA was analyzed by agarose (1%) gel electrophoresis. DNA was detected using ethidium bromide (Fisher BioReagents). A Kodak gel documentation system (EDAS 290, Kodak, Rochester, NY) was used to capture digital images of the gels.

### Release study

For release studies, pelleted spheres were resuspended in 2 mL ddH_2_O. Separate samples for each time point were prepared by diluting 150 μL of sphere suspension (containing approximately 750 μg of zein and 4.3 μg of DNA) in 3 mL of release media. These samples were incubated in a humidified 37°C chamber for varying times. At predetermined time points, an entire sample was removed from the incubator and spheres were separated from the supernatant by centrifugation. The spheres and the supernatant were extracted with phenol/chloroform and DNA was quantified by Hoechst assay, both as described above.

DNA release from spheres into 1X PBS was measured over 7 days. Samples for loaded and blank spheres were centrifuged at 0, 1, 3, 6, 9, 12, 24, 48, 72, 96, 120, 144, and 168 hours. At each time point, samples were centrifuged at 10,000 g for 30 minutes at room temperature to separate spheres from the supernatant and analyzed as described above. Release was calculated:

(4) $$PBS\;Release=\frac{Mass\;of\;DNA\;in\;Supernatant\left(at\;Time=t\right)}{Total\;Mass\;of\;DNA\left(at\;Time=0\right)}\times 100\%$$

### Cellular response

#### Cell culture

Human embryonic epithelial kidney cells, HEK 293T (ATCC, Manassas, VA), were cultured in T-75 flasks in Dulbecco’s modified Eagle’s medium (DMEM, Gibco/Invitrogen, Carlsbad, CA) containing 4.5 g/L glucose and supplemented with 10% fetal bovine serum (FBS, Gibco), 2 mM L-glutamine (Gibco), and 1% penicillin/streptomycin (Gibco). For seeding, cells were counted using a hemocytometer and trypan blue staining for viable cells after being dissociated with 1 mM EDTA. Human colon carcinoma cells, Caco-2, were obtained from ATCC and were cultured in T-75 flasks in Eagle’s minimum essential medium (EMEM, ATCC) supplemented with 20% FBS (Gibco) and 1% penicillin/streptomycin (Gibco). For seeding, cells were dissociated with 0.05% trypsin/EDTA (Gibco). For both cell lines, cells were seeded into 48-well plates or 8-well Nunc Lab-Tek chambered coverglass slides (Thermo Scientific) at a density of 30,000-33,000 cells per well for HEK 293T cells and 25,000 cells per well for Caco-2 cells.

#### Cytotoxicity

Cytotoxicity was assessed using a Water Soluble Tetrazolium (WST-1) salts cell proliferation assay kit (Roche, Indianapolis, IN). For the WST-1 assay, nanospheres containing 0.1, 0.25, 0.5, 1.0 or 2.0 μg of DNA were formed at a zein to DNA ratio of 80:1, resuspended in 1 mL ddH_2_O, and then diluted in OptiMem (Gibco) to a final volume of 75 μL. Cells were seeded in 48-well plates as described above and after 24 hours of culture, zein/DNA nanospheres were added to the culture media in triplicate wells. Cells were imaged to observe cell morphology (Leica DMI 3000B, Bannockburn, IL) and the WST-1 assay was conducted according to the manufacturer’s protocol at 24, 48, and 72 hours after the addition of zein nanospheres. Briefly, cells were washed with PBS, and incubated at 37°C with the WST-1 solution (10 vol.% WST-1 reagent in phenol-free DMEM, 400 μl /well). After incubation for 3 hours, absorbance values of WST-1 solution (100 μl from each well) were measured on an Epoch Microplate spectrophotometer (BioTek, Winooski, VT) at a wavelength of 430 nm. Assays were performed in triplicate on duplicate days.

#### Confocal microscopy

To verify that confocal microscopy could be used to image zein/DNA nanospheres without labeling, the autofluorescence of zein was investigated. The autofluorescence of various zein concentrations in 70% ethanol was measured in a fluorometer with ultraviolet and blue modules. Once autofluorescence was confirmed, confocal microscopy was used to assess cellular morphology in response to the zein nanospheres, as well as to analyze cellular association of the spheres. Caco-2 and HEK 293T cells cultured with 80:1 or 250:1 nanospheres were imaged using a confocal microscope (Olympus IX 81, Olympus, Center Valley, PA). Cells were seeded as described above into 8-well coverslides. Zein spheres were visualized with an excitation wavelength of (405 nm) and an emission wavelength of (590 nm) and these images were overlaid with corresponding phase images.

#### Sphere internalization

To assess internalization of zein nanospheres, Caco-2 cells were seeded onto 8-well coverslides described above and after 36 hours of culture, media was replaced and zein/DNA nanospheres containing 0.1 μg DNA at a zein to DNA ratio of 80:1 were added to the fresh culture media. Twelve hours after the media change and addition of spheres, the cells were washed and then stained using Vybrant DiO cell-labeling solution (Molecular Probes/Life Technologies, Carlsbad, CA) for 25 minutes at 37° C according to the manufacturer’s protocol. Cells were then washed with PBS and fixed with 10% buffered formalin for 10 minutes. Following staining, confocal microscopy was performed, with zein and DiO sequentially excited using dual excitation of 405 and 543 nm for zein and 488 nm for DiO. A series of 15 optical sections in the Z-plane were acquired at intervals of 1 μm for at least two different locations per well and images were processed using Olympus FluoView software (v.5.0) to determine location of the spheres relative to cellular membranes.

### Statistics

All experiments were performed between three and six times (noted in figure legends). Comparative analyses were completed using a student’s t-test or one-way ANOVA followed by Tukey’s multiple comparison test for multiple data points, both at a 95% confidence level using Prism software (GraphPad Prism 5, LaJolla, CA). Mean values with standard error of the mean are reported for all data.

## Competing interests

The authors declare that they have no competing interests.

## Authors’ contributions

MCR participated in design of the study and carried out all studies on encapsulation, size, and release and drafted the manuscript. JDT carried out the cytotoxicity, DNase I protection assays, internalization studies and assisted in manuscript preparation and editing. TB carried out a portion of the DLS studies. YY participated in the design of the study. AKP conceived of the study, and participated in its design and coordination and helped to draft the manuscript. All authors read and approved the final manuscript.

## Supplementary Material

Additional file 1 ** Figure S1. **Size, PdI, and zeta potential for nanospheres resuspended at various salt concentrations (A, B, C respectively), formed at 80:1 zein to DNA ratio. One hour data labeled with the same letter do not vary significantly while asterisks in the legend denote a significant difference (p < 0.05) between zero and one hour measurements. All data are reported as mean ± standard error of the mean, with n = 3. Click here for file

Additional file 2 ** Figure S2. **Autofluorescence of zein at various concentrations using ultraviolet (365–395 nm) and blue (465–485 nm) modules. Click here for file
