# Supplementary figures and images for: Fabrication and characterization of DNA-loaded zein nanospheres
Source: J Nanobiotechnology. 2012 Dec 2;10:44. doi: 10.1186/1477-3155-10-44 (PMC3524772; doi:10.1186/1477-3155-10-44)

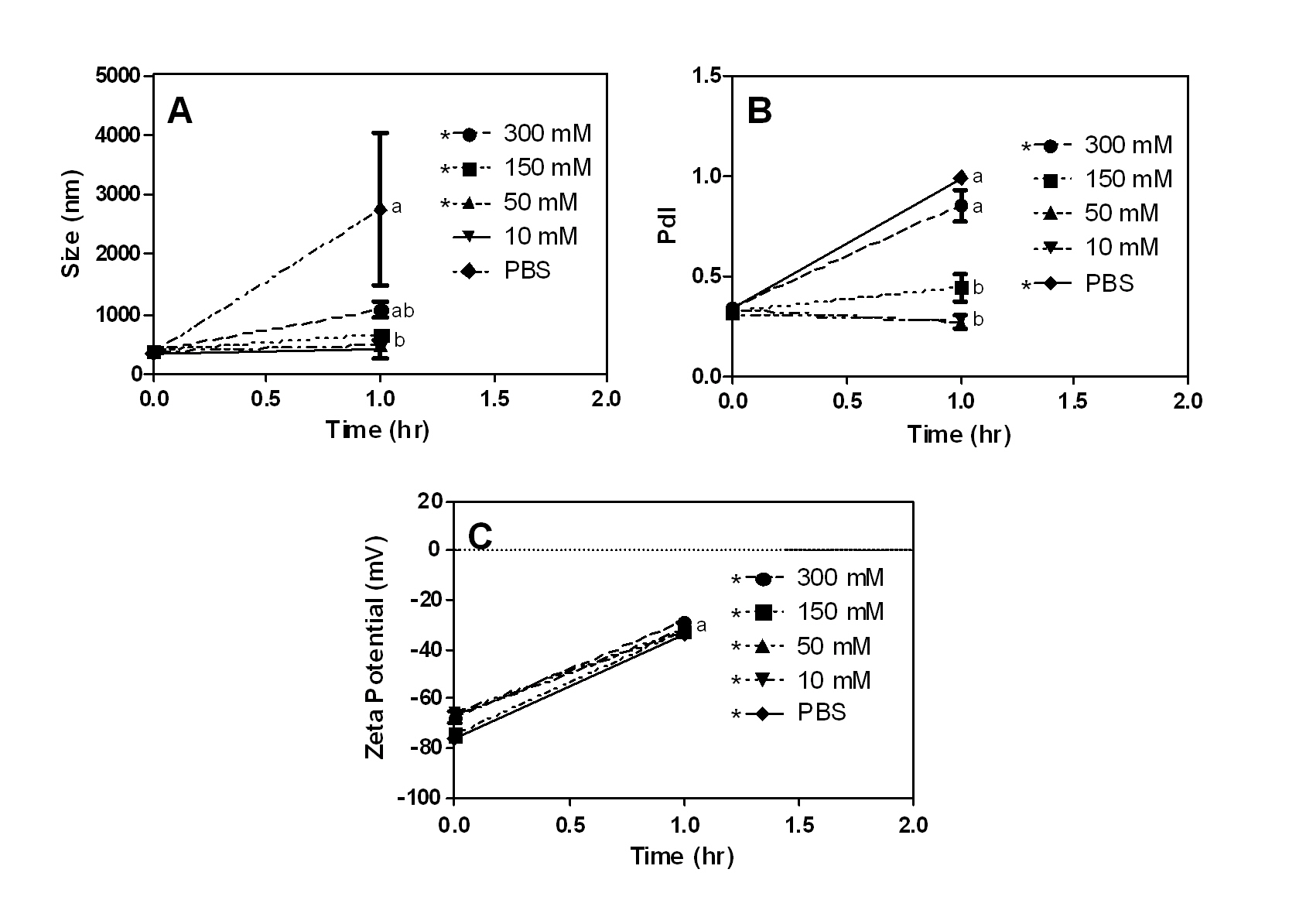

Supplement: Additional file 1 — Figure S1. Size, PdI, and zeta potential for nanospheres resuspended at various salt concentrations (A, B, C respectively), formed at 80:1 zein to DNA ratio. One hour data labeled with the same letter do not vary significantly while asterisks in the legend denote a significant difference (p < 0.05) between zero and one hour measurements. All data are reported as mean ± standard error of the mean, with n = 3. [file 1477-3155-10-44-S1.jpeg]

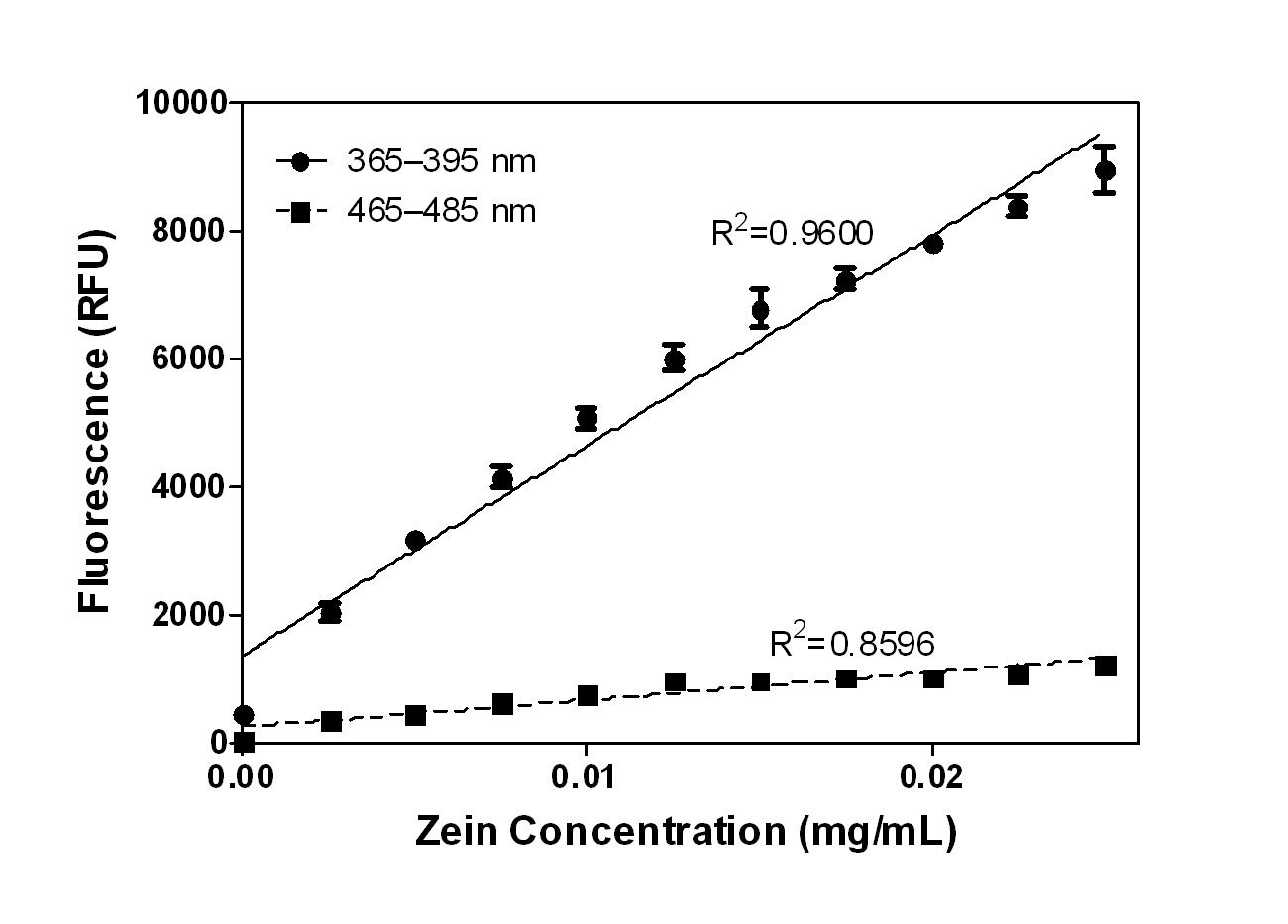

Supplement: Additional file 2 — Figure S2. Autofluorescence of zein at various concentrations using ultraviolet (365–395 nm) and blue (465–485 nm) modules. [file 1477-3155-10-44-S2.jpeg]
